# Mucus Impaction Related to Postoperative Anastomosis Site Obstruction: A Rare Case

**DOI:** 10.7759/cureus.58048

**Published:** 2024-04-11

**Authors:** Fu-Fei Yang, Ren-Hao Chan

**Affiliations:** 1 Department of Surgery, National Cheng Kung University Hospital, College of Medicine, National Cheng Kung University, Tainan, TWN; 2 Division of Colorectal Surgery, Department of Surgery, National Cheng Kung University Hospital, College of Medicine, National Cheng Kung University, Tainan, TWN

**Keywords:** complication, laparoscopic surgery, complication management, colonoscope, laparoscopic colon resection

## Abstract

Anastomotic stricture has an incidence rate of 6-10% and typically manifests three to six months after colorectal surgery. Immediate postoperative stricture is exceedingly rare and underreported in the literature. The possible etiology includes poor circulation, leakage, local inflammation, or infection. We report a rare case of a patient with total obstruction by mucus on the anastomosis site on postoperation day two. We used a sigmoidoscope to remove mucus material, following which the patient recovered well.

## Introduction

Anastomotic stricture is a prevalent complication following colorectal surgery involving the use of a circular stapler [1]. The primary symptom often includes difficulty passing stool or gas, typically occurring three to six months after the operation. Symptomatic stricture necessitating intervention is observed in only 6%-10% of patients [2]. Immediate postoperative stricture is exceptionally rare, with a different etiology than ischemia or leakage, requiring time for stricture formation to develop. Current understanding of the etiology of anastomosis stricture is limited, and the well-known presentation of stenosis is stricture regarding the possible cause and risk factors. We report a rare case of postoperative mucus impaction on the anastomosis site after laparoscopic surgery, which led to total colon obstruction.

## Case presentation

A 54-year-old female patient presented to our outpatient clinic for a rectal tumor incidentally found on an abdominal CT scan during a health examination. She reported no dizziness, bowel habit changes, constipation, bloody or blood-tinged stools, and abdominal pain. The patient was referred to our clinic for further evaluation regarding the need for surgical intervention.

During a colonoscopy, a large, soft tumor was observed at 30-38 cm from the anal verge around the descending sigmoid junction; hence, a colonic lipoma was suspected. The lower gastrointestinal series was similar to the colonoscopy findings, except for an intussusception at the sigmoid colon that reduced after an air enema. Subsequently, the patient received bowel preparation with 4 L split-dose polyethylene glycol, followed by laparoscopic anterior resection with end-to-end anastomosis with a circular stapler.

**Figure 1:**
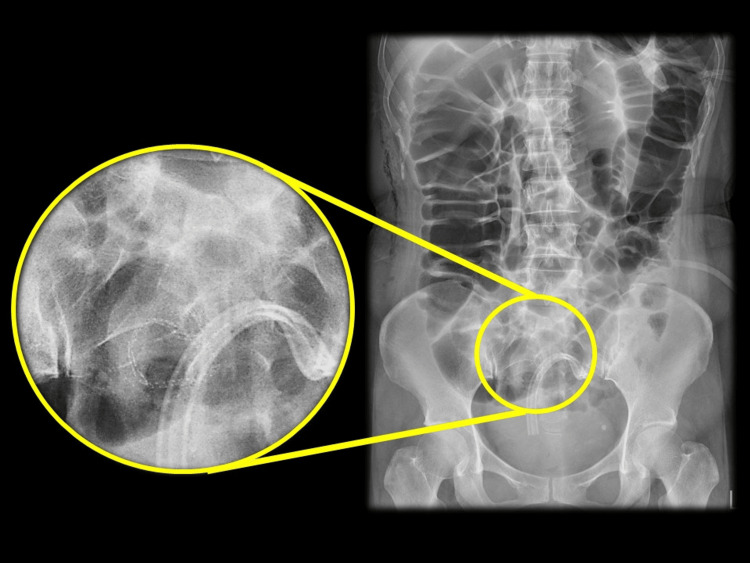
The abdominal X-ray. The abdominal X-ray revealed a significant amount of bowel gas originating from the small bowel to the descending colon and ended in anastomosis.

On postoperative day two, the patient complained of mild abdominal distention and cramping pain after drinking water. The symptoms worsened in the following days and became intolerable. An abdominal X-ray revealed a significant amount of bowel gas originating from the small bowel, diminishing in the anastomosis (Figure 1).

**Figure 2:**
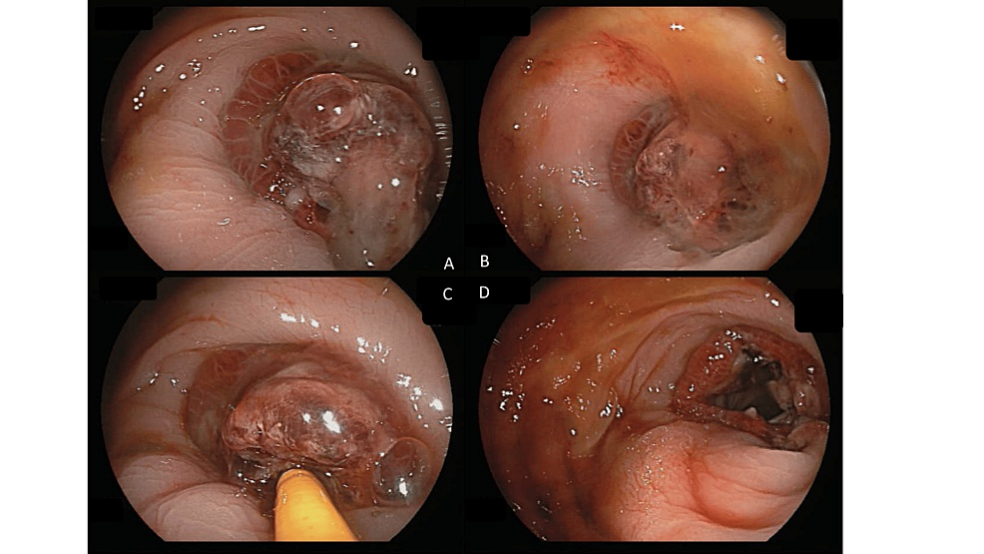
Colonoscopic examination. (A, B) Colonoscope showing mucus material at the anastomosis site leading to total obstruction. (C) The mucus was removed with a forceps through the colonoscope. (D) The patent anastomosis after the procedure.

Owing to persistent symptoms, an emergent colonoscopy was conducted on postoperative day six, which revealed a mucus-based object occupying the entire lumen, causing bowel obstruction at the anastomosis site (above anal verge 15 cm). After the removal of the mucus content, the anastomosis and further colon lumen were patent (Figure 2).

**Figure 3:**
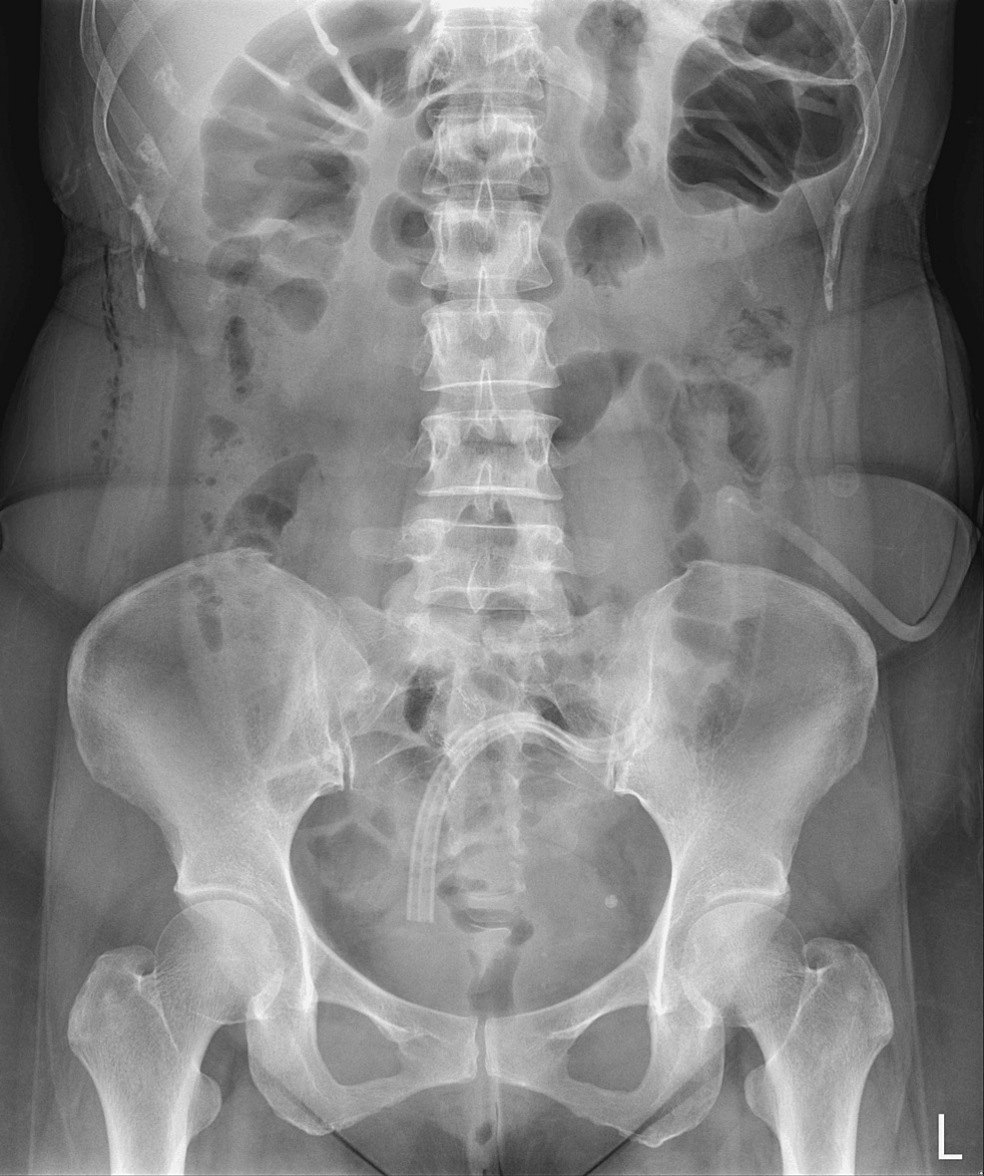
Post-procedure abdominal X-ray. The abdominal X-ray after the sigmoidoscope showed much improvement in colon gas, with the gas passing through anastomosis to the rectum.

The patient experienced improvement on the same day. Furthermore, the radiograph obtained the following day showed improvement (Figure 3).

**Figure 4:**
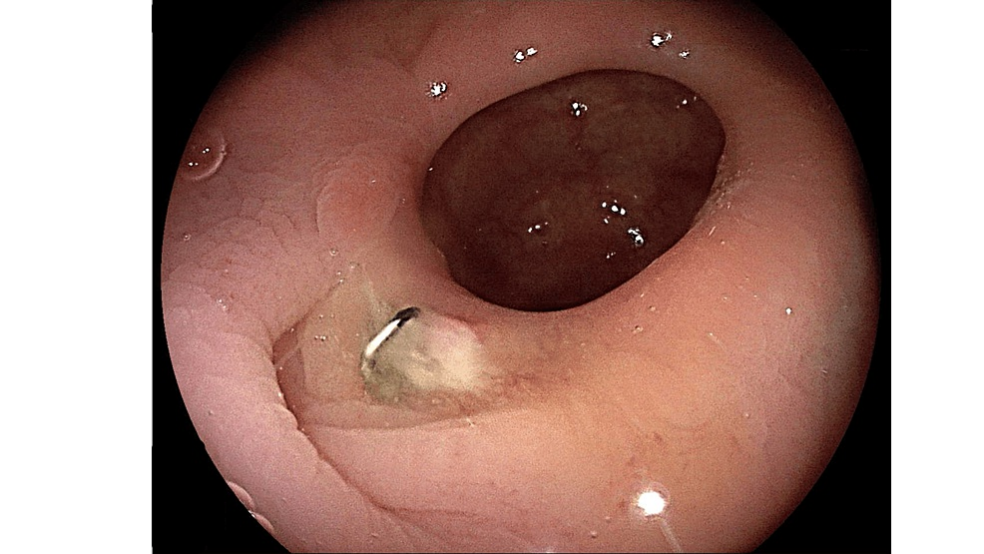
Follow-up colonoscopy. Follow-up colonoscopy after six months. The well-healed and patent anastomosis.

The patient’s hospital course was uneventful. She was discharged with a full oral diet. The pathology results revealed a sigmoid colon lipoma. After discharge, the patient reported smooth defecation at home. Six months later, a follow-up colonoscopy showed favorable healing of the anastomosis (Figure 4).

## Discussion

In colorectal surgery, circular staple anastomosis has long been an established procedure. Anastomosis stricture is a common complication following colorectal surgery, with a reported incidence of up to 30% under endoscopic assessment postoperatively, while most patients remain asymptomatic [2,3]. Ambrosetti et al. have reported symptomatic anastomosis stricture following elective laparoscopic sigmoidectomy, with an incidence of 17.6% [4]. The pathophysiology and risk factors of anastomosis stricture are poorly understood; however, anastomosis stricture is associated with the anastomotic technique, tissue tension over anastomosis, anastomosis leakage, use of circular stapler, blood circulation, digestive tract obstruction, infection, neoadjuvant chemoradiotherapy, and general nutrition status [4-6]. While some anastomosis strictures may improve spontaneously, symptomatic ones usually require treatment, with endoscopic intervention being the preferred treatment [2,7]. Other factors contributing to stricture encompass insufficient circular staple size, local tumor recurrence, or bowel adhesion [1,2,4]. Goblet cells play a crucial role in synthesizing and secreting gut mucus to safeguard the underlying epithelium. The continuous renewal of mucus is a highly active process. Preoperative antibiotic colon preparation can alter the microbiota, affecting mucus secretion and potentially causing damage. Surgical anastomosis, another form of trauma, can also trigger excessive mucus secretion [8]. Previous studies have reported postoperative anastomosis of colonic anastomosis by scar, managed with colonoscopic intervention [9]. We report a rare case of mucus impaction on anastomosis resulting in bowel obstruction in which colonoscopic removal of mucus material was successfully performed, and the anastomosis remained intact after the procedure. Colonoscopy provides diagnostic value and helps resolve stenosis in the perioperative period.

## Conclusions

Our case presents a unique etiology and presentation timeline compared to the previous literature. It is the first documented instance of postoperative anastomosis stenosis attributed to mucus. Various diagnostic and management tools are available, with the colonoscope being a particularly effective diagnostic instrument capable of promptly addressing obstructive material. Fortunately, our patient’s recovery was uneventful, and no long-term sequelae were observed.
